# Effect of endotoxin absorption by polymixin B hemoperfusion in dialysis-requiring acute kidney injury

**DOI:** 10.1186/2197-425X-3-S1-A625

**Published:** 2015-10-01

**Authors:** M Iwagami, Doi, H Yasunaga, E Noiri, N Yahagi

**Affiliations:** University of Tokyo, Tokyo, Japan

## Introduction

Endotoxin absorption treatment using polymixin B direct hemoperfusion (PMX-DHP) has been clinically applied to septic shock patients for the last two decades in Japan. The total number of filter usage appears to be more than 200,000. However, no clinical trial has been conducted with sufficient statistical power so far.

## Objectives

By using a Japanese national inpatient database, the survival benefit of PMX-DHP in septic shock patients complicated with acute kidney injury (AKI) requiring continuous renal replacement therapy (CRRT).

## Methods

Adult patients in the Japanese Diagnosis Procedure Combination Database satisfying the following criteria were enrolled: hospitalized during 2007-2012; started CRRT in ICUs; diagnosed as having sepsis; and required noradrenaline or dopamine. Propensity scores for receiving PMX-DHP were generated based on patient demographics, background conditions, treatment regimens in addition to hospital characteristics.

## Results

Of 3,759 eligible patients, 1,068 received PMX-DHP. Propensity score matching produced a matched cohort of 1,005 pairs. The 28-day mortality was 40.4% in the PMX-DHP group and 46.9% in the control group (P = 0.003). Logistic regression analysis revealed a significant association between the use of PMX-DHP and decreased 28-day mortality (adjusted odds ratio, 0.74; 95% confidence interval, 0.62-0.90).

## Conclusions

This large retrospective study using a Japanese nationwide database demonstrates septic shock complicated with severe AKI requiring CRRT might benefit from PMX-DHP.

## Grant Acknowledgment

The Ministry of Health, Labour and Welfare, Japan (Research on Policy Planning and Evaluation grant number H22-Policy-031), Ministry of Education, Culture, Sports, Science and Technology, Japan (Scientific Research B, No. 22390131), Council for Science and Technology Policy, Japan (Funding Program for World-Leading Innovative R&D on Science and Technology, FIRST program-grant number 0301002001001).

